# Notes and News

**Published:** 1897-02-20

**Authors:** 


### NOTES AND NEWS.

(Collected and Communicated.)

### HOSPITAL MEETINGS.

The annual court of the Dreadnought Hospital, Greenwich, will be held at the Royal United Service Institution, on Thursday, the 25th inst., at three p.m.

The annual meeting of governors of the West London Hospital will be held in the new building, on Wednesday, March 3rd, at five p.m.

The annual meeting of governors of Queen Charlotte's Lying-in Hospital will be held at the Hospital, on Monday, February 22nd, at half-past three p.m.

The anniversary dinner of the German Hospital will be held on April 30th, at the Hôtel Métropole, the Duke of Cambridge presiding.

The anniversary dinner of the London Orphan Asylum, Watford, will be held on March 1st, at the Hôtel Métropole. The Earl of Verulam will preside.

The annual meeting of the Great Northern Central Hospital will be held at the hospital, on Friday, the 26th inst., at five p.m.

The Indian Famine Fund has reached upwards of £297,000.

The Dublin Hospital Sunday Collection amounted to £4,388 this year.

A good many cases of small-pox have recently appeared in Hong Kong, especially amongst Europeans. The Government are taking steps to stamp out the disease.

A festival dinner in aid of the funds of the City of London Hospital for Diseases of the Chest will be held at the Hotel Metropole on Tuesday, March 23rd, the Hon. W. F. D. Smith in the chair.

The Duchess of Teck opened the new wards at the Walthamstow Hospital last week. She has consented that one of the wards shall be named after her. Mr. Courtney Warner, president of the hospital, has contributed £1,000 to the funds.

The Coroner at a recent inquiry held at St. George's-in-the-East respecting the death of a child from diphtheria, was requested by the jury to forward their expressed opinion to the Asylums Board that the Board was to blame in the matter. It was stated at the inquest that the child was removed from the London Hospital to its home by the Asylums Board, under the plea that they had no accommodation for it.

The employés of Messrs. Marshall and Snelgrove gave an entertainment on behalf of the Middlssex Hospital last week, whi ch was most successful.

Mr. Bancroft, by his readings, which he intends to resume next winter with the same object, has made gifts to various hopitals in London and the provinces of sums exceeding £3,000.

Bedfordshire is to have a new County Hospital. The cost is estimated at £33,000. Towards this £27,000 has already been subscribed, of which sum the Duke of Bedford and Mr. Whitbread each gave £5,000.

There is great excitement at Cape Town owing to the report which is circulated that Dr. Koch has discovered a rinderpest anti-toxin. The Government is arranging for a series of lectures by Dr. Koch to veterinary experts.

Affairs at the Radcliffe Infirmary continue most unsatisfactory. The executive and medical staff appear unable to agree as to the administration, and Lord Dillon and the Rev. C. Fletcher have both resigned their connection with the institution.

The Protest Committee, who are the active opponents of the vivisection movement, forwarded to the Home Office a memorial against the British Institute of Preventive Medicine being registered for experiments on living animals. The petition bore the signatures of 183,706 London residents.

The Mercers' Company have granted £1,000 towards the special appeal for £100,000 now being made on behalf of Charing Cross Hospital. The Leathersellers' Company have granted £100, the Brewers' Company £21, and the Cutlers' Company and Scriveners' Company £10 10s. each towards the same fund.

Dr. Thorne Thorne, of the Local Government Board; Mr. H. Farnall, of the Foreign Office; Surgeon-General Richardson, of the Indian Medical Service; and Professor Notter, of Netley Hospital, have gone to Venice to attend, on behalf of Great Britain, the International Conference on the bubonic plague. Dr. Notter is a new appointment, and his business is to watch matters in connection with transport of troops. The other British delegates are the Hon. Robert Herbert and Surgeon-General Cleghorn, Indian Medical Department.

If we accept the statistics of the plague in Bombay as worked out by the *Times of India*, we must regard the position of affairs as even more appalling than the official reports which have reached England have led us to suppose. There seems much probability that many cases of plague are not recognised as such, and are registered under other heads, thus explaining the high general mortality which has coincided with the onset of the plague. The journal referred to has taken the death-rate of corresponding weeks during the past five years, and argues that the number of deaths above the average may fairly be taken to relate to victims from the plague. According to this mode of reckoning, the mortality from the disease reaches about ten thousand since its first recognised appearance to the last week in January, and is three times as large as officially reported.

